# The Effects of Dyslipidemia in Subclinical Hypothyroidism

**DOI:** 10.7759/cureus.6173

**Published:** 2019-11-16

**Authors:** Azhar Hussain, Abdelfatah M Elmahdawi, Noor El-Hudda Elzeraidi, Fatimah Nouh, Khalid Algathafi

**Affiliations:** 1 Medicine, Xavier University School of Medicine, Oranjestad, ABW; 2 Lab Medicine, Higher Institute of Medical Professions, Benghazi, LBY; 3 Biochemistry, Faculty of Medicine, University of Benghazi, Benghazi, LBY

**Keywords:** dyslipidemia, low-density lipoprotein cholesterol, subclinical hypothyroidism, thyroid stimulating hormone (tsh), total cholestrol

## Abstract

Background

Subclinical hypothyroidism (SCH) affects 7.5-8.5% of women and 2.8-4.4% of men globally. Usually, both hypothyroidism and hyperthyroidism are related to cardiovascular and cerebrovascular disease development. The relationship between subclinical hypothyroidism and dyslipidemia has been widely investigated, but the findings remain controversial. Recent evidence shows that serum thyroxine (T4) replacement therapy may improve lipid profiles. The objective of the present study is to assess dyslipidemia among patients with SCH in Benghazi, Libya and compare it with controls.

Methods

The study was conducted from August 2018 to November 2018 and included 36 patients with SCH. All the patients were around 30 years of age. We also included sex-matched healthy subjects (controls) selected from three diabetes and endocrinology clinics in Benghazi: Alhaya clinic, Alrazy clinic, and Alnukbah clinic. Clinical information and medical history were obtained through a questionnaire from all SCH patients and normal control subjects. Blood samples were collected and analyzed for thyroid-stimulating hormone (TSH), free thyroxine (FT4), total cholesterol (T-Chol), serum triglycerides (STG), low-density lipoprotein-cholesterol (LDL-C), and high-density lipoprotein-cholesterol (HDL-C).

Results

Patients with SCH showed significantly higher T-Chol, STG, and LDL-C levels, as well as significantly lower levels of HDL-C in comparison to the healthy controls. No significant correlation was found between TSH and T-Chol, STG, HDL-C, and LDL-C; no significant correlation was found between FT4 and HDL-C either. However, a strong negative correlation was found between FT4 and T-Chol, STG, and LDL-C.

Conclusion

Our study concluded that SCH is associated with dyslipidemia. We strongly recommend biochemical screening for thyroid dysfunction for all patients with dyslipidemia.

## Introduction

Subclinical hypothyroidism (SCH) is characterized by high levels of serum thyroid-stimulating hormone (TSH) along with normal levels of serum thyroxine (T4) and triiodothyronine (T3) with few or no signs/symptoms of hypothyroidism [1]. The discussions about the management of subclinical thyroid dysfunction have been controversial. The prevalence of SCH is about 4-8.5% globally and maybe as high as 20% in women older than the age of 60 years [2]. There is good evidence that SCH can often progress to overt disease. SCH is a strong indicator of the risk for atherosclerosis and myocardial infarction in elderly women [3]. SCH is associated not only with elevated low-density lipoprotein-cholesterol (LDL-C) levels and low high-density lipoprotein-cholesterol (HDL-C) levels but also with elevated lipoprotein(a). This may further increase the risk of the development of atherosclerosis [4].

## Materials and methods

Subjects

The study was conducted from August 2018 to November 2018 and included 36 patients with SCH. All the patients were around 30 years of age. We also included sex-matched healthy subjects (controls) selected from three diabetes and endocrinology clinics in Benghazi: Alhaya clinic, Alrazy clinic, and Alnukbah clinic. Patients suffering from any disease other than SCH that could affect their metabolic status and the parameters studied, such as previous or family history of thyroid disorders, malignancy, liver disease, kidney disease, acute or chronic inflammation, recent surgery, diabetes, or any disease that could affect patient lipid profiles were excluded from the study. Pregnant and lactating women were also excluded.

Methods

The tests for total cholesterol (T-Chol), serum triglycerides (STG), HDL-C, and LDL-C were done using the standard procedures and available commercial kits in a fully automated system, COBAS INTEGRA 400 plus (Roche, Germany). TSH and free thyroxine (FT4) were measured using COBAS e 411 (Roche, Germany) by electrochemiluminescence technique.

Statistical Analysis

The data were analyzed using the statistical package for the social sciences (SPSS) Windows version 17 (IBM, Armonk, NY). Descriptive characteristics of the study participants were calculated as mean (±) standard deviation (SD). An independent sample t-test was used to determine the differences in subject characteristics. Pearson’s correlation coefficient determination was done to evaluate the degree of association between thyroid function changes and clinical and biochemical parameters. A p-value (two-tailed) of <0.05 was considered as statistically significant.

## Results

The mean age and SD of SCH patients selected for this study were [42.8 (±13.4)], and the male-to-female ratio was 13:23. The age range was 19-63 years. The mean age and SD of the healthy control subjects were [44.8 (±12.0)], and the male-to-female ratio was 12:18. The age range was 24-68 years.

The mean of TSH was significantly higher in SCH patients [13.60 (±11.81) mIU/L] when compared to normal controls [2.03 (±0.65) mIU/L] (p: 0.00). The FT4 mean concentration was significantly lower in SCH [12.91 (±2.61) ng/dl] than normal healthy controls [16.20 (±1.21) ng/dl] (p: 0.00). The level of T-Chol was even markedly higher in SCH patients [215.08 (±38.75) mg/dl] than in normal healthy controls [174.50 (±29.15) mg/dl] (p: 0.00). The mean level of STG was significantly higher in SCH patients [153.58 (±50.86) mg/dl] when compared to normal healthy controls [130.26 (±40.74) mg/dl] (p: 0.00). However, the mean level of HDL-C was significantly lower in SCH patients [25.83 (±6.86) mg/dl] when compared to normal healthy controls [37.86 (±3.96) mg/dl] (p: 0.00). The mean level of LDL-C was significantly higher in SCH patients [129.97 (±20.97) mg/dl] in contrast to normal healthy controls [89.93 (±10.45) mg/dl] (p: 0.00). All relevant parameters are laid out in Table 1.

**Table 1:** Comparison of parameters between subclinical hypothyroidism patients and the control group SCH: subclinical hypothyroidism; n: number; TSH: thyroid-stimulating hormone; FT4: free thyroxine; T-Chol: total cholesterol; STG: serum triglycerides; HDL-C: high-density lipoprotein-cholesterol; LDL-C: low-density lipoprotein-cholesterol *Figures in parentheses represent standard deviation

Parameters	SCH patients, n = 36	Controls, n = 30	P-value
TSH, mIU/L	13.60 (±11.81)*	2.03 (±0.65)*	0.00
FT4, ng/dl	12.91 (±2.61)*	16.20 (±1.21)*	0.00
T-Chol, mg/dl	215.08 (±38.75)*	174.50 (±29.15)*	0.00
STG, mg/dl	153.58 (±50.86)*	130.26 (±40.74)*	0.00
HDL-C, mg/dl	25.83 (±6.86)*	37.86 (±3.96)*	0.00
LDL-C, mg/dl	129.97 (±20.97)*	89.93 (±10.45)*	0.00

**Figure 1:**
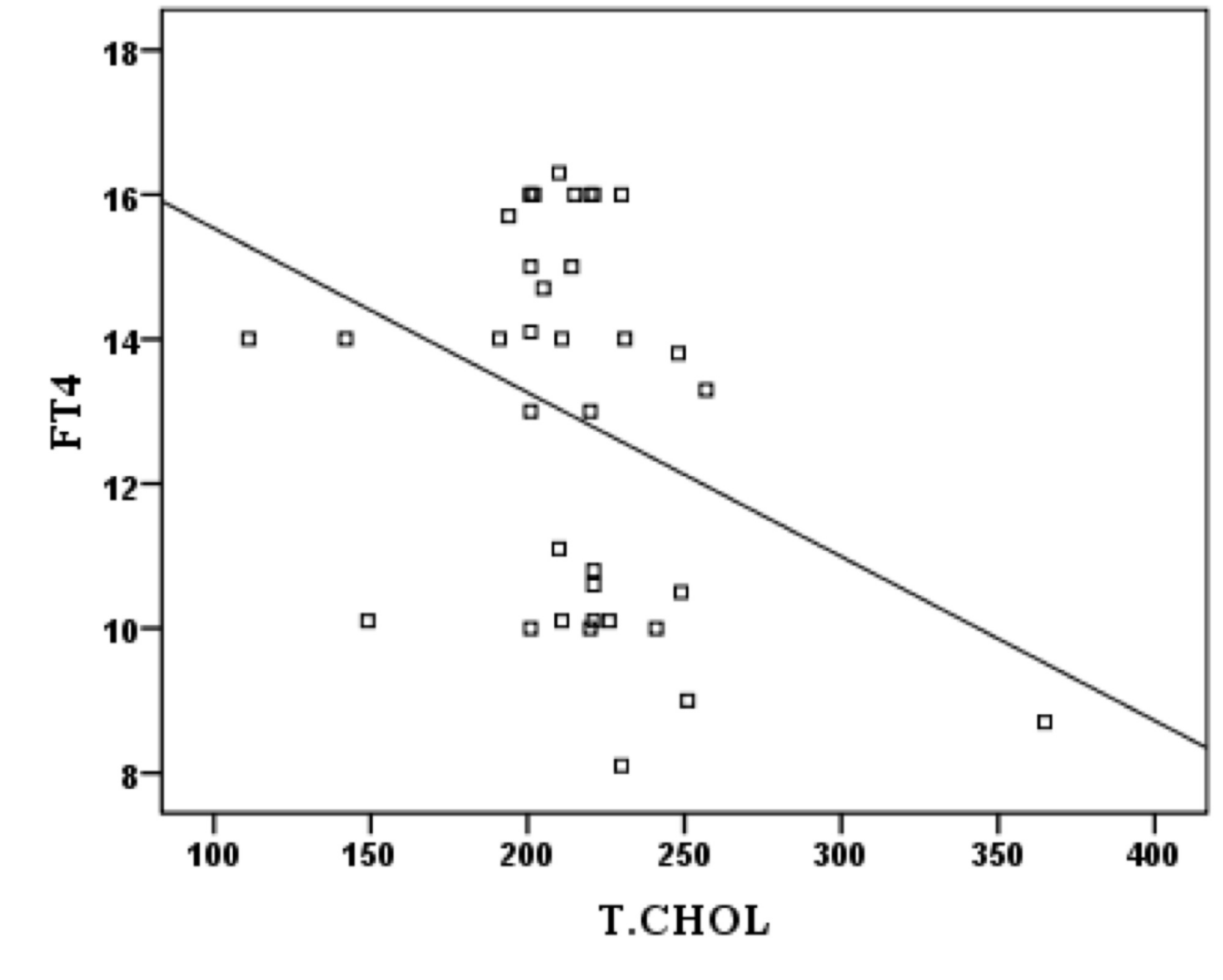
Correlation between FT4 and T-Chol in subclinical hypothyroidism patients FT4: free thyroxine; T-Chol: total cholesterol

**Figure 2:**
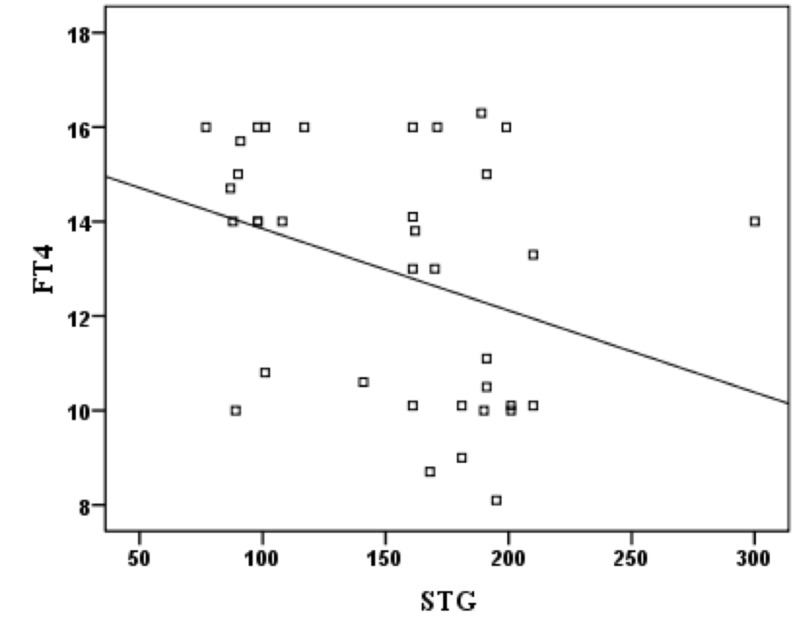
Correlation between FT4 and STG in subclinical hypothyroidism patients FT4: free thyroxine; STG: serum triglycerides

**Figure 3:**
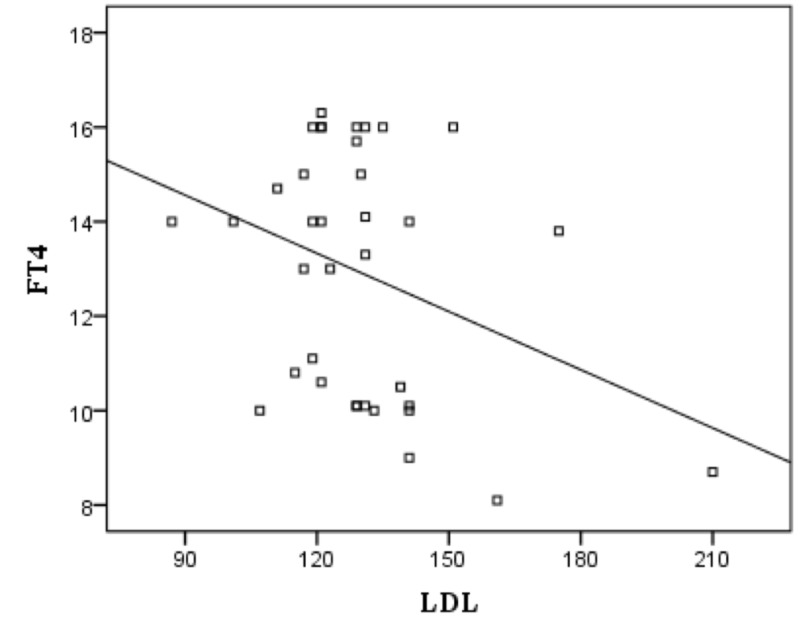
Correlation between FT4 and LDL-C in subclinical hypothyroidism patients FT4: free thyroxine; LDL-C: low-density lipoprotein-cholesterol

There was no significant correlation between TSH and T-Chol, STG, HDL-C, and LDL-C. And no significant correlation was found between FT4 and HDL-C. However, there was significant negative correlation between FT4 and T-Chol (p: 0.044, r: -0.337) (Figure 1), STG (p: 0.044, r: -0.338) (Figure 2) and LDL-C (p: 0.049, r: -0.330) (Figure 3).

## Discussion

This study shows a strong connection between SCH and dyslipidemia. Dyslipidemia was more established in patients with SCH in comparison to the control group, and the levels of T-Chol, STG, and LDL-C were significantly higher among SCH patients in comparison to controls. HDL-C levels were significantly lower in SCH patients in comparison to controls. This finding is in agreement with the study by Hueston et al. as it also showed an increase in T-Chol levels in patients with SCH in contrast with the control group. But, after the reconciliation of confounding elements and the use of cholesterol-lowering drugs, no significant difference was observed between the two groups regarding the lipid profile [5]. In a study conducted by Lai et al., low levels of thyroid hormone in euthyroid patients were associated with dyslipidemia in a Chinese community [6]. A study by Luboshitzky et al. found that among the lipid profiles of SCH patients, only STG levels were higher when compared to the control group [7]. Another study conducted by Al Sayed et al. showed that T-Chol and LDL-C levels were significantly higher in patients with SCH compared to the control group [8]. However, there are many studies that did not find this positive relationship between SCH and dyslipidemia [9,10], which contrasts with the results of our study.

## Conclusions

In conclusion, dyslipidemia and its dangerous effects on the cardiovascular system, including diastolic dysfunction, ischemic heart disease (IHD), heart failure, and an overall increase in mortality imply that SCH is considered an atherogenic condition as it causes an increase in lipid levels and an increased risk for cardiovascular disease. Therefore, biochemical screening for thyroid dysfunction is recommended for all patients with dyslipidemia and underlying hypothyroidism. Treatment of SCH with thyroxine improves quality of life and decreases the risk for dyslipidemia and cardiovascular disease.
